# Non-Alcoholic Fatty Liver Disease: The Bile Acid-Activated Farnesoid X Receptor as an Emerging Treatment Target

**DOI:** 10.1155/2012/934396

**Published:** 2011-12-07

**Authors:** Michael Fuchs

**Affiliations:** Division of Gastroenterology, Hepatology and Nutrition, Department of Internal Medicine, Virginia Commonwealth University School of Medicine, P.O. Box 980341, Richmond, VA 23298-0341, USA

## Abstract

Non-alcoholic fatty liver disease (NAFLD) is currently evolving as the most common liver disease worldwide. It may progress to liver cirrhosis and liver cancer and is poised to represent the most common indication for liver transplantation in the near future. The pathogenesis of NAFLD is multifactorial and not fully understood, but it represents an insulin resistance state characterized by a cluster of cardiovascular risk factors including obesity, dyslipidemia, hyperglycemia, and hypertension. Importantly, NAFLD also has evolved as independent risk factor for cardiovascular disease. Unfortunately thus far no established treatment does exist for NAFLD. The bile acid-activated nuclear farnesoid X receptor (FXR) has been shown to play a role not only in bile acid but also in lipid and glucose homeostasis. Specific targeting of FXR may be an elegant and very effective way to readjust dysregulated nuclear receptor-mediated metabolic pathways. This review discusses the body's complex response to the activation of FXR with its beneficial actions but also potential undesirable side effects.

## 1. Introduction

One characteristic of our modern civilization is the easy and unlimited access to unhealthy and caloric dense food. A typical American diet furnishes the liver with ~20 g of fat each day, equivalent to one-half of the total triglyceride content of the liver. In combination with little need for physical activity due to technological advances, one consequence of our sedentary and excessive lifestyle is non-alcoholic fatty liver disease (NAFLD).

NAFLD is a major health problem affecting up to 60 million Americans and evolving as the most common liver disease worldwide [1, 2]. This is several-fold higher than other common chronic liver diseases such as hepatitis C and alcohol-related liver disease. While the majority of subjects with NAFLD are obese, the condition can occur in the absence of obesity or other features of the metabolic syndrome. In patients with diabetes and morbid obesity the prevalence of NAFLD has been shown to be as high as 62% and 96%, respectively [3, 4].

The earliest stage of NAFLD is fatty liver that is defined as the presence of cytoplasmic triglyceride droplets in more than 5% of hepatocytes [5]. Although often self-limited, in 12–40% it can progress to non-alcoholic steatohepatitis (NASH) [6]. NASH is distinguished from simple fatty liver by the presence of hepatocyte injury such as hepatocyte ballooning and apoptosis, an inflammatory infiltrate, and/or collagen deposition. Over a time period of 10–15 years, 15% of patients with NASH will progress to liver cirrhosis [7]. Once cirrhosis has developed in the absence of viral hepatitis, hepatic decompensation occurs at a rate of 4% annually while the ten-year risk of developing liver cancer is 10% [7, 8]. Although liver cancer secondary to NASH typically develops in the setting of cirrhosis, carcinogenesis can occur in the absence of advanced liver disease. It is thus not surprising that NAFLD is poised to become the primary indication for liver transplantations. Like other causes of chronic liver disease, NASH recurs following liver transplantation almost universally [9].

## 2. Basic Pathophysiological Concepts and Treatment of NAFLD

**Figure 1:**
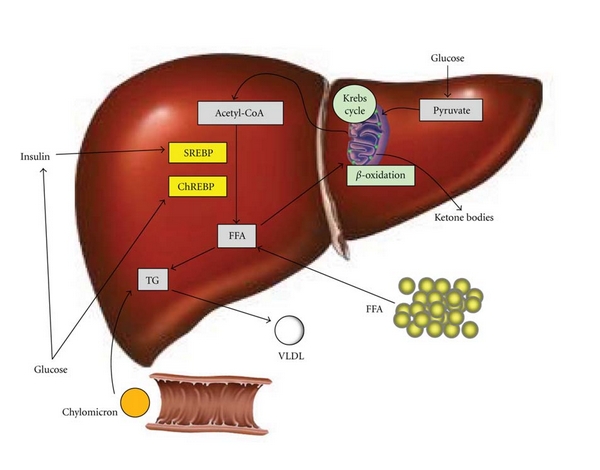
Hepatic triglyceride (TG) formation, acquisition, and removal. Fatty liver is a result of an imbalance between free fatty acids (FFAs), and TG input and FFA and TG output. FFA derives from peripheral tissue, endogenous synthesis or diet in form of chylomicrons. Carbohydrate intake increases glucose and insulin levels thereby promoting lipogenesis through the activation of transcription factors sterol regulatory element-binding protein 1c (SREBP) and carbohydrate responsive element-binding protein (ChREBP). Reducing the FFA burden include *β*-oxidation in mitochondria, storage as TG, or export as very low-density lipoprotein (VLDL).

The pathogenesis of NAFLD is multifactorial and only partially understood. Fatty liver arises in the setting of an imbalance between triglyceride formation/acquisition and removal (Figure 1). Assembly of triglycerides and lipid droplet formation requires fatty acids that can derive from diet, *de novo* synthesis, or adipose tissue. Dietary fat packed in chylomicrons is hydrolyzed releasing free fatty acids of which approximately 20% are delivered to the liver [8]. Carbohydrate-enriched diets promote *de novo* synthesis of free fatty acids via insulin-stimulated activation of sterol regulatory element-binding protein-1c [10, 11]. In addition, glucose facilitates lipogenesis via activation of carbohydrate responsive element-binding protein [12]. In the fasting state, a decline of insulin levels stimulates adipocyte triglyceride hydrolase thereby releasing free fatty acids that are transported to the liver [13]. In the liver, free fatty acids can be (i) used for energy and ketone body production via mitochondrial *β*-oxidation, (ii) esterified and stored as triglycerides in lipid droplets, or (iii) packaged with apolipoprotein B into very low-density lipoproteins that are secreted into the circulation. As the liver extracts approximately 20% of free fatty acids from the circulation, the daily input of triglycerides from diet and fatty acids from adipocyte tissue is equivalent to the entire triglyceride content of the liver [14]. Once the capacity of the liver to store fatty acids in form of triglycerides is overwhelmed, NASH, differentiated from a fatty liver by the presence of increased cell injury, apoptosis, inflammation, and fibrosis, starts to develop. A detailed review of the steps involved in the progression of NAFLD to NASH and cirrhosis has been recently published [15].

Treatment of NAFLD should either prevent disease progression to liver cirrhosis or reverse already established NASH, respectively. Despite many advances in our understanding of the pathogenesis of NAFLD, there is currently no established treatment available. Life-style changes and exercise to reduce body weight and treatment of concomitant diabetes and dyslipidemia are accepted first-line therapy but have not been shown to convincingly reduce the risk of disease progression [16]. Therefore exploring new avenues for treatment of this common disease is crucial.

## 3. The Bile Acid-Activated Nuclear Farnesoid X Receptor (FXR)

Nuclear receptors are a group of transcription factors that consist of 48 members in humans. They have a common structure consisting of a ligand-independent activation domain for interaction with cofactors, a central DNA binding domain, and a unique ligand binding domain allowing receptor dimerization and coregulator interactions. Most nuclear receptors function either as homodimers or as heterodimers with the retinoid X receptor. Binding of the ligand promotes conformational changes facilitating the release of corepressors and resulting in conformational changes of chromatin enabling access of the transcriptional machinery to the respective promoters. Upon ligand activation, the corepressor complex dissociates and the coactivator complex is recruited allowing start of transcription. Control of nuclear transcriptional activity is also thought to occur by posttranslational modifications [17–19].

In 1995 a protein was discovered that was interacting with the human retinoic X receptor and named retinoic X receptor-interacting protein 14 [20]. Because it was activated by an intermediate of the mevalonate pathway, farnesol, it was renamed to farnesoid X receptor [21]. Another four years later, three independent groups [22–24] discovered bile acids as endogenous ligands for FXR. From an evolutionary point of view the *FXR* gene is highly conserved suggesting that it plays an important role in many species. At the tissue level, FXR is expressed predominantly in the liver, intestine, kidney, and adrenal gland. Expression in heart and adipose tissue is low [25]. The generation of mice with *Fxr* gene ablation identified FXR as a master regulator in bile acid homeostasis [26]. Subsequently novel functions of FXR have been identified including protecting the intestinal barrier and modulating the innate immunity [27, 28] and tumorigenesis [29, 30]. The most important roles of FXR are likely in regulating metabolic processes.

## 4. FXR as Key Player in Multiple Metabolic Processes

For a long time, physiological effects of bile acids have mainly been attributed to their physicochemical properties [31]. In the last couple of years it has been evident that bile acids act like signaling molecules [32] regulating not only their own homeostasis during the enterohepatic circulation but also triglyceride, cholesterol, and glucose metabolism.

### 4.1. Bile Acid Metabolism

A major physiological role of FXR in bile acid metabolism is to protect hepatocytes from the deleterious effects of increased bile acid levels by inhibiting endogenous bile acid synthesis and accelerating bile acid biotransformation and excretion. In this regard, FXR-mediated effects occur in a tightly coordinated fashion at the level of the hepatocyte and enterocyte and have been reviewed in detail elsewhere [33].

### 4.2. Triglyceride and Cholesterol Metabolism

It has been known for years that bile acids can modulate lipid metabolism in humans. Reducing the transhepatic flux of bile acids decreases low-density lipoprotein cholesterol and increases high-density lipoprotein cholesterol and very low-density lipoprotein triglyceride levels. Opposite effects are observed when the bile acid pool is expanded [34–36]. Studies in mice with *Fxr* gene ablation or administering FXR agonists provided key information demonstrating a central role of FXR in lipid homeostasis.

**Figure 2:**
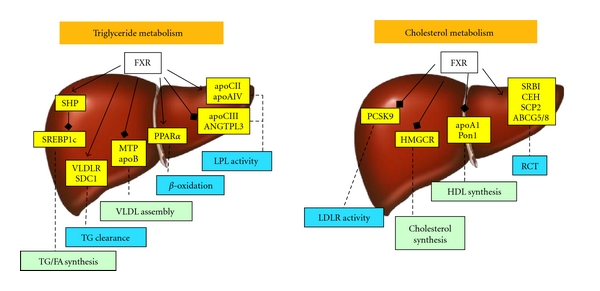
Effect of FXR activation on triglyceride and cholesterol metabolism in the liver. FXR agonists result in a variety of responses modulating triglyceride (TG) and cholesterol metabolism. Activation of FXR inhibits triglyceride (TG)/fatty acid (FA) synthesis facilitated by suppressing sterol regulatory element-binding protein 1c (SREBP1c) via activation of short heterodimer partner (SHP). FXR controls assembly of very low-density lipoprotein (VLDL). FXR may increase the clearance of TG by stimulating lipoprotein lipase (LPL) activity as well as the hepatic uptake of remnants and low-density lipoprotein by inducing syndecan 1 (SDC1) and the VLDL receptor (VLDLR). FXR agonists may modulate LDL receptor activity via inhibition of proprotein convertase subtilisin/kexin 9 (PCSK9) and activate the reverse cholesterol transport pathway (RTC). FXR activation also impairs high-density lipoprotein (HDL) formation and suppresses cholesterol synthesis. apoA1, apoB, apoCII, apoCIII, apoAIV: apolipoprotein A1, B, CII, CIII, AIV; ANGTPL3: angiopoetin like 3; ABCG5/8: adenosine triphosphate binding cassette subfamily G member 5/8; CEH: cholesterylester hydrolase; HMGCR: 3-hydroxy-3-methylglutaryl coenzyme A reductase; MTP: microsomal triglyceride transfer protein; PON1: paraoxonase 1; SRBI: scavenger receptor B1; SCP2: sterol carrier protein 2.

 As illustrated in Figure 2, FXR activation of short heterodimer partner is required to suppress sterol regulatory element-binding protein 1c expression [37]. As sterol regulatory element-binding protein 1c is known to regulate several genes involved in fatty acid and triglyceride formation [11], FXR-mediated repression of sterol regulatory element-binding protein 1c inhibits triglyceride and fatty acid synthesis and secretion. Interestingly, recent studies support the concept that FXR-independent mechanisms may also contribute [38]. In addition to decreasing lipogenesis, activation of FXR facilitates the clearance of very low-density lipoproteins and chylomicrons. This is achieved by increasing the expression of the very low-density lipoprotein receptor [39], a protein that enhances lipoprotein lipase-mediated triacylglycerol hydrolysis. Very low-density lipoprotein assembly is controlled by FXR via repressing the expression of microsomal triglyceride transfer protein and apolipoprotein B [38]. FXR also activates syndecan-1, a transmembrane protein that binds remnant particles before their transfer to receptors [40]. Activation of lipoprotein lipase, a key enzyme involved in the lipolysis of triglyceride rich lipoproteins, is also FXR-dependent. This involves activation of apolipoproteins C-II and AIV [41–43] and inhibiting the expression of apolipoprotein C-III [44] and angiopoetin-like 3 [37], respectively. Another effect of FXR activation is the induction of peroxisome proliferator-activated receptor *α* that promotes fatty acid *β*-oxidation [45]. Collectively these findings support the concept that FXR activation decreases plasma triglyceride levels by suppressing hepatic lipogenesis and triglyceride secretion and increasing the clearance of triglyceride-rich lipoproteins from blood. These observations therefore support the concept that FXR activation may have a beneficial effect in patients with NAFLD by decreasing hepatic lipogenesis. 

Activation of FXR also modulates the reverse cholesterol transport, a pathway that promotes cholesterol delivery from the periphery to the liver for biliary disposal and fecal elimination. In this scenario, the selective uptake of high-density lipoprotein cholesteryl ester via scavenger receptor BI [46], intracellular cholesteryl ester hydrolysis facilitated by neutral cholesteryl ester hydrolase [47], as well as the canalicular routing of cholesterol by sterol carrier protein 2 [48] for biliary excretion via adenosine triphosphate binding cassette subfamily G member 5/8 [49] are positively regulated by FXR [50]. In addition but controversial, FXR appears to suppress apolipoprotein A-I expression [46, 50, 51], the primary protein constituent of high-density lipoprotein defining its size and shape. This may be of particular importance as it could influence the capability of high-density lipoprotein to remove cholesterol from peripheral cells, activating the lecithin-cholesterol acyl transferase enzyme and delivering the resulting cholesteryl ester to the liver. Another target of FXR is paraoxonase 1, a protein produced in the liver with phospholipase A2 activity that may be important for inactivation of proatherogenic lipids produced by oxidative modification of low-density lipoprotein. FXR-mediated repression of paraoxonase 1 involves the induction of fibroblast growth factor 19, its subsequent binding to the fibroblast growth factor receptor 4, and activation of the c-Jun N-terminal kinase pathway [52, 53]. FXR also regulates the expression of phospholipid transfer protein [54] that is responsible for the transfer of phospholipids and cholesterol from low to high-density lipoprotein and suppresses 3-hydroxy-3-methyl-glutaryl-CoA reductase likely involving sterol regulatory element-binding protein 2 [55]. Finally, FXR represses proprotein convertase subtilisin/kexin 9 [56], a protein that promotes the intracellular degradation of the low-density lipoprotein receptor by interfering with its recycling to the plasma membrane. In summary, these findings raise concern that activation of FXR may alter the cholesterol metabolism in a way that increases the susceptibility to atherosclerosis and thus limit its application in patients with NAFLD.

### 4.3. Glucose Homeostasis

In addition to their pleiotropic effects on lipid metabolism, bile acids also affect glucose homeostasis. This is supported by an improved glycemic control in patients with diabetes mellitus response to cholestyramine [57]. Several studies addressed the role of bile acids and FXR activation in glucose metabolism, but the underlying mechanisms are far from being understood. It appears clear that FXR exerts a role in glucose homeostasis [58]. In the state of *Fxr* gene ablation, the failure to suppress gluconeogenesis and a reduced peripheral glucose disposal led to glucose intolerance [59–61]. A potential molecular basis for these observations is the suppression of hepatic phosphenoyl-pyruvate carboxykinase and glucose 6-phosphatase [60, 62]. Reduced plasma levels of free fatty acids in response to FXR activation (see above) may explain the increased insulin sensitivity in the liver. Of note, FXR activation was shown to enhance insulin-stimulated glucose uptake as well as insulin signaling in adipocytes [61]. It should be noted that bile acids also modulate glucose homeostasis in an FXR-independent fashion through cell signaling pathways [63]. Collectively these findings suggest that FXR activation might prove useful in the treatment of hyperglycemia and hyperlipidemia that are present in patients with NAFLD.

### 4.4. Hepatic Inflammation and Fibrogenesis

Inflammation and collagen deposition in the liver are key histopathological features of NASH. FXR appears to antagonize hepatic inflammatory processes by antagonizing the nuclear factor kappa B pathway [64]. Another protective FXR mechanism involves induction of antimicrobial factors in the intestine [65]. As FXR is expressed in rodent hepatic stellate cells that play a critical role in hepatic fibrosis, it is not surprising that FXR agonists protect against liver fibrosis [66]. This appears to be mediated by a decreased hepatic expression of various profibrotic growth factors including transforming growth factor *β*1, tissue inhibitor of metalloproteinase 1, *α*1(I) collagen, *α* smooth muscle actin, matrix metalloproteinase 2 and *α*2(I) collagen, and microRNA-29a [67–69]. However, if this mechanism is also operational in humans with a lower expression level of FXR remains to be determined [70]. These data suggest that targeting FXR may impact progression from NAFLD to NASH.

## 5. FXR and Atherosclerosis

**Figure 3:**
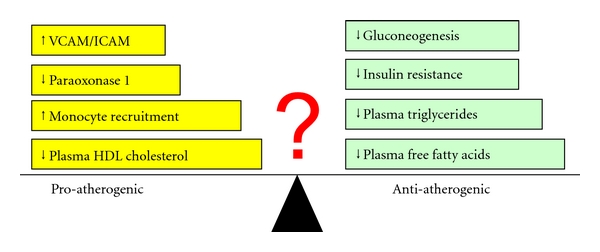
Pro- and antiatherogenic effects of FXR activation. With regard to atherosclerosis, activation of FXR may be associated with beneficial and potential negative effects. Unless tested in humans, one cannot predict with certainty whether pro- or antiatherogenic effects are dominant and development of specific FXR modulators may help to avoid some or most of the negative effects. VCAM: vascular cell adhesion protein. ICAM: intracellular adhesion molecule.

As demonstrated earlier in this article and illustrated in Figure 3, activation of FXR seems to be associated with both anti- and proatherogenic properties. In addition to its impact on dyslipidemia and hyperglycemia, FXR may also directly act at the levels of the arterial wall. Potential beneficial effects of FXR activation against atherosclerosis include suppressing the vasoconstrictive peptide endothelin-1 [71]. Induced expression of intracellular adhesion molecule 1 and vascular cell adhesion molecule 1, however, promotes atherosclerosis by recruiting macrophages to the endothelium [72]. The role of FXR in the initiation and progression of atherosclerosis has been studied in mice with *Fxr* gene ablation that were backcrossed into atherosclerosis-susceptible strains with either deletion of the low-density lipoprotein receptor or apolipoprotein E, respectively [73, 74]. These studies produced discrepant results whereas more recent experimentations employing an FXR agonist uniformly demonstrated protection against diet-induced aortic plaque formation [75, 76]. Translating these findings to humans is not straightforward as humans carry most cholesterol in LDL compared to the mouse that lacks cholesteryl ester transfer protein activity and thus transports most cholesterol in high-density lipoprotein [77]. In knowledge of these limitations, it would be most logical to carry out future studies in low-density lipoprotein receptor deficient mice that overexpress human cholesteryl ester transfer protein [78].

## 6. Summary and Perspective

FXR plays a key role in the transcriptional control of a myriad of target genes that control metabolic pathways relevant to NAFLD. By virtue of that role FXR is critically involved in the development and progression of NAFLD. Targeting FXR therefore offers exciting new perspectives for the treatment of NAFLD. However, when interpreting data obtained in cell culture and rodent models of human disease, attention needs to be paid to differences between these models and humans. One particular challenge in designing FXR agonists is separating the desired therapeutic effects from the undesirable side effects. The design of organ- or gene-specific FXR ligands may enhance the specificity and reduce side effects of this therapeutic approach. An increased understanding of the effect of cellular signaling of FXR and its coregulator proteins has the potential to aid in discovering novel selective therapeutic modulators and the development of new and more effective therapeutics. Finally one also needs to consider that the response to modulation of the FXR receptor may differ among patient with NAFLD and NASH.

Despite all the concerns raised, it is anticipated that targeting FXR will result in a more specific and individually tailored therapy that could revolutionize the management of NAFLD. Support comes from studies in rats with diabetes mellitus and fatty liver disease that received the FXR agonist INT-747 for two months [79]. This intervention decreased glucose levels and dyslipidemia, protected against body weight gain, and improved insulin resistance. It is thus very encouraging that INT-747 also has shown to improve insulin resistance in patients with diabetes mellitus and NAFLD [80]. Based on this study with a limited number of patients, an ongoing large multicenter trial enrolling 280 patients at eight U.S. centers comprising the NIDDK-sponsored NASH Clinical Research Network is under way, the results of which are eagerly awaited.
